# FOLFIRI^®^ and Bevacizumab in first-line treatment for colorectal cancer patients: safety, efficacy and genetic polymorphisms

**DOI:** 10.1186/1756-0500-7-260

**Published:** 2014-04-23

**Authors:** Yves Bécouarn, Laurent Cany, Marina Pulido, Richard Beyssac, Patrick Texereau, Valérie Le Morvan, Dominique Béchade, René Brunet, Sofiane Aitouferoukh, Caroline Lalet, Simone Mathoulin-Pélissier, Marianne Fonck, Jacques Robert

**Affiliations:** 1 Department of Digestive Oncology, Institut Bergonié, Comprehensive Cancer Centre, 229 cours de l’Argonne, F-33000 Bordeaux, France; 2 Polyclinique Francheville, Périgueux, France; 3 Clinical and Epidemiological Research Unit, Institut Bergonié, Comprehensive Cancer Centre, 229 cours de l’Argonne, F-33000 Bordeaux, France; 4 CIC-EC7 (Clinical Investigation Centre – Clinical Epidemiology), Bordeaux, France; 5 Maison de Santé Protestante Bagatelle, Talence, France; 6 Centre Hospitalier Layné, Mont de Marsan, France; 7 INSERM U916, Institut Bergonié, Comprehensive Cancer Centre, 229 cours de l’Argonne, F-33000 Bordeaux, France; 8 Univ. Bordeaux, F-33000 Bordeaux, France

**Keywords:** Bevacizumab, Chemotherapy, Clinical trial, phase II, Colorectal neoplasms, FOLFIRI® protocol

## Abstract

**Background:**

Over 50% of colorectal cancer (CRC) patients develop metastases. The aim of this study was to evaluate efficacy and tolerance of first-line FOLFIRI® + bevacizumab (B) treatment for metastatic CRC, and to assess genetic polymorphisms as potential markers.

**Methods:**

Adult patients with histologically-proven, non-resectable metastatic CRC and ECOG ≤ 2 were included. 14-day cycles consisted of bevacizumab (5 mg/kg), irinotecan (180 mg/m^2^), bolus FU (400 mg/m^2^) and leucovorin (400 mg/m^2^), followed by 46-hour FU infusions (2400 mg/m^2^). Primary endpoint was response rate according to RECIST criteria. Secondary endpoints were overall (OS) and progression-free (PFS) survivals, response duration, and toxicity. Associations between clinical data, *UGT1A1*, thymidylate synthase, *VEGFA* polymorphisms and PFS, OS and toxicity were analyzed.

**Results:**

Sixty-two patients were enrolled (median age 68y). 59/62 patients were eligible and evaluable for response at 6 months: 28 showed partial response (47.5%; 95% CI; 34.3-60.9), 20 stable disease (33.9%) and 11 progression (18.6%). Grade 3/4 toxicities were as follows: neutropenia 16.1%; diarrhea 11.3%; nausea-vomiting 1.6%. Median response duration was 9.5 months (range 2.7-20); median PFS 10.3 months (range 8.8-11.7); and median OS 25.7 months (range 20.2-29.7). 11/59 initially unresectable patients were resectable after treatment. *VEGFA* polymorphism (rs25648) was associated with better OS (HR: 3.61; 95% CI: 1.57-8.30).

**Conclusions:**

FOLFIRI® + bevacizumab is active with good response rate, long median OS, and a good safety profile. A VEGFA polymorphism might have a prognostic value in this malignancy.

**Trial registration:**

Clinicaltrials.gov: NCT00467142 (registration date: April 25, 2007)

## Background

Colorectal cancer (CRC) is a major public health problem. Its incidence in France is increasing [1] with approximately 40 000 new cases per year [2] and prognosis remains poor [3]. Over 50% of patients will develop metastases and will be candidates for palliative chemotherapy [4]. Bevacizumab is a monoclonal antibody directed against the vascular endothelial growth factor (VEGF). It has proven efficacy in the treatment of metastatic CRC when combined with chemotherapy [5-7]. Irinotecan, infusional 5-fluorouracil (FU), leucovorin (LV) (FOLFIRI®) and bevacizumab (FOLFIRI® + B) offered better outcomes when compared to irinotecan plus infusional fluorouracil (FU)/leucovorin (LV) (FOLFIRI®), irinotecan plus bolus FU/LV (mIFL), and irinotecan plus oral capecitabine (CapeIRI) in a randomized trial [5]. However, a relatively high rate of ≥ Grade 3 hypertension was observed.

Several gene polymorphisms may interfere with anticancer drug activity, and thus affect drug efficacy and toxicity. For FU, a thymidylate synthase promoter 28-bp tandem repeat (rs34743033) is associated with lower efficacy and increased toxicity [8]. For irinotecan, a *UGT1A1* promoter TA repeat (rs8175347) is a risk factor for toxicity [9]. For Bevacizumab, several *VEGFA* single nucleotide polymorphisms (SNP) are known to influence *VEGFA* plasma concentrations [10] and to be associated with CRC risk [11]. In addition, prognostic [12,13] and predictive [14,15] roles of *VEGFA* variants have been identified in various studies.

The principal objective of this phase II trial was to evaluate efficacy of first-line treatment with FOLFIRI® + B for metastatic CRC patients in terms of response rates. Secondary objectives were to assess overall and progression-free (PFS) survivals, response duration, and toxicity. We also explored common gene polymorphisms known to interfere with the metabolism and/or activity of FOLFIRI® + B, located respectively in the thymidylate synthase (*TYMS*), UDP-glucuronosyltransferase 1A1 (*UGT1A1*) and *VEGFA* genes, looking for associations between these polymorphisms and the clinical parameters of toxicity and efficacy of the treatment.

## Methods

Patients for this open-label, single arm, phase II trial were recruited from Institut Bergonié, the University Hospital of Bordeaux, and five general hospitals and private clinics in South-West France.

Inclusion criteria were: histopathologically-proven adenocarcinoma of the colon or rectum, non-resectable metastatic disease; no prior chemotherapy other than adjuvant chemotherapy (provided it had been discontinued > 6 months before study entry); Eastern Cooperative Oncology Group (ECOG) performance status ≤ 2; age ≥ 18 years; measurable metastatic disease per Response Evaluation Criteria in Solid Tumors (RECIST Version 3.0) [16]; adequate hematological function [hemoglobin ≥10 g/dl, absolute neutrophils count ≥1.5 × 10^9^/l, platelets ≥100 × 10^9^/l]; adequate renal function [no proteinuria and creatinine ≤1.25 × the upper limit of the normal value (ULN)]; adequate hepatic functions [total bilirubin ≤1.25 × ULN, aspartate amino-transferase (AST) and alanine aminotransferase (ALT) ≤3 × ULN, in case of liver metastases, total bilirubin ≤1.5 × ULN and AST and ALT ≤5 × ULN]; intervals since inclusion of 4 weeks for eventual surgery or radiotherapy; ability to comply with scheduled follow-up and management of toxicity.

Exclusion criteria included: histology other than adenocarcinoma; non-measurable disease; adjuvant chemotherapy within 6 months or containing Bevacizumab; unresolved bowel or partial bowel obstruction; history of chronic diarrhea; severe gastrointestinal toxicity while receiving FU; current uncontrolled infection; serious illness or medical condition; previous abdominopelvic radiation therapy; known Gilbert’s syndrome; arterial thromboembolism accident or myocardial infarction within preceding 6 months; history of cancer other than colorectal, except for curatively treated non-melanoma skin cancer or in-situ cervical cancer; concomitant treatment with any other investigational drug; and pregnancy.

The protocol was approved by the regional Ethics Review Committee (Comité de Protection des Personnes du Sud-Ouest et d’Outre-Mer) and registered with clinicaltrials.gov (NCT00467142, registration date April 25, 2007). Each patient provided written informed consent. For the complementary pharmacogenetic study, patients were enrolled on a voluntary basis and gave specific consent.

### Treatment

FOLFIRI® + B treatment consisted of a 90-min I.V. infusion of bevacizumab (5 mg/kg) followed by a 90-min I.V. infusion of irinotecan (180 mg/m^2^) followed by a simplified LV5FU2 regimen [leucovorin (400 mg/m^2^) and bolus fluorouracil (400 mg/m^2^) on day 1 and a 46-h infusion of fluorouracil (2400 mg/m^2^)]. Treatment was delivered biweekly.

FOLFIRI® doses were adjusted in the event of toxic effects (National Cancer Institute Common Terminology Criteria for Adverse Events, NCI–CTCAE, version 3.0 [17], according to the following guidelines. Hematological toxicity was evaluated during each cycle. In the event of myelosuppression (i.e. absolute neutrophils count <1.5 × 10^9^/l and/or platelets <75 × 10^9^/l) at the planned date for the next cycle of chemotherapy, treatment was postponed for 1 to 3 weeks until recovery. After a 4-week delay with no recovery, the patient left the study. In the event of recovery, the FU bolus at day 1 was deleted if the toxicity was related to the neutrophils count. The continuous FU infusion at day 1 and 2 was reduced by 25% if the toxicity was related to the platelet count. The same dose reductions were implemented in the event of grade 4 neutropenia or thrombocytopenia, or grade 3 neutropenia associated with fever. After two dose reductions, patients left the study. In the event of grade 3 or 4 diarrhea, the irinotecan dose was reduced to 150 mg/m^2^ and the FU bolus was deleted at day 1. In the event of a second episode of severe diarrhea, the continuous FU infusion was reduced by 25%. In the event of grade 3 or 4 mucositis or hand-foot syndrome, a 25% dose reduction of FU bolus and FU continuous infusion was carried out.

Bevacizumab dose was not reduced. For severe drug-induced toxicities, treatment was stopped, either temporarily or indefinitely. In the case of gastrointestinal perforation, grade 3 or 4 hemorrhage, thromboembolic accidents, severe hypertension or grade 4 proteinuria, bevacizumab was stopped indefinitely.

### Assessment methods

Pre-inclusion work-up included an initial radiologic assessment within the 3 weeks before treatment onset, and clinical and biological evaluations conducted in the week before inclusion. The first administration occurred within 8 days of inclusion. During treatment, clinical and biological assessments were conducted on day 1 of each 14-day cycle. Radiologic assessment was carried out every four cycles (8 weeks) with centralized external secondary review. Treatment toxicity was evaluated before each cycle (NCI–CTCAE v3). Treatment was discontinued in the event of disease progression, unacceptable toxicity or patient refusal. Patients were followed-up every 3 months after treatment discontinuation.

### Genotyping

DNA was extracted from blood samplings obtained after patient inclusion and collection of informed consent. We used the kit QIAamp® DNA purchased from Qiagen according to the instructions of the manufacturer. Genotyping of DNA extracts was performed using a customized platform, SNPChip484, enabling simultaneous determination of 384 selected SNP from a DNA extract. It consists of a collection of kits containing the primers specific for each SNP, prepared upon demand by Illumina and used according to the BeadXpress Goldengate-Veracode technology. Rough genotyping results were treated and analyzed using the Genome Studio software from Illumina, which enables the individual determination for each DNA sample of the genotype of all 384 SNPs. Although we genotyped 384 different SNPs, we analyzed only the results concerning the three *VEGFA* polymorphisms that were scheduled in the protocol: rs699947 (-2578C > A), rs2010963 (-634G > C), rs25648 (S178S, formerly known as -7C > T) in order to avoid statistical problems associated with multiple testing.

### Genotyping of UGT1A1 and TYMS

The variations in the TYMS gene were determined using RFLP techniques [18]. The TA repeat in the UGT1A1 promoter (UGT1A1-28 genotype, rs8175347) was determined by pyrosequencing performed after PCR amplification with the following primers: sense: 5′GAACTCCCTGCTACCTTTGTG3′), antisense (biotinylated): 5′TTTGCTCCTGCCAGA GGTT3′. PCR products were analyzed without further purification on a Pyrosequencer PyroMark ID system (Qiagen, Courtaboeuf, France) according to the instructions of the manufacturer with the following sequencing primer: 5′ TCGATTGGTTTTTGC3′. The SQA mode was used to analyze the TA repeat. The variations in the TYMS gene were determined using RFLP techniques as follows: for the 3′UTR insdel polymorphism (rs16430), PCR was performed using the following primers: sense: 5′- CAAATCTGAGGGAGCTGAGT-3′; antisense: 5′-CAGATAAGTGGCAGTACAGA-3′. The PCR products were digested by DraI, which specifically cleaves the +6 allele, and subjected to polyacrylamide gel electrophoresis. For the 5′UTR tandem repeat variation in the TYMS gene promoter (rs34743033), PCR was performed using the following primers: sense: 5′-AGGCGCGCGGAAGGGGTCCT-3′; antisense: 5′-TCCGAGCCGGCCACAGGCAT-3′. The 2R/3R variation was first identified by direct electrophoresis of the PCR products on 12% polyacrylamide gels; the PCR products were then digested by HaeIII, which specifically cleaves the 3G allele, and subjected to polyacrylamide gel electrophoresis.

### Statistical considerations

A two-stage Simon’s design was used. Using unacceptable and acceptable response rates of 50% and 70% respectively, a 5% type I error rate and a 10% type II error rate (90% power), the total sample size for this trial was 61 assessable patients over two stages, with 24 assessable subjects recruited during the first stage. At the end of the first stage, 14 PR/CR were required to continue. At the end of the second stage, 37 PR/CR were required to conclude efficacy.

The primary endpoint of this study was the response rate (RR) at 6 months (both partial [PR] and complete [CR]), evaluated according to RECIST as reviewed by an independent expert committee. Secondary endpoints were progression-free survival (PFS), response duration, overall survival (OS) and toxicity. PFS was calculated from the time of inclusion to disease progression or death of any cause, and duration of the time of the documented response to the progression date. OS was calculated from the first treatment cycle to death (of any cause). PFS and OS were calculated by the Kaplan-Meier method. Median follow-up was calculated with the reverse Kaplan-Meier method. Univariate analyses were performed to determine factors associated with higher OS, PFS and toxicity from clinical and pharmacogenetic data. A Fisher’s exact test was used to evaluate the association of investigated genotypes, clinical data and toxicity. The associations between genotypes, clinical data and survival were tested using the log-rank test. The effects on OS and PFS were estimated by hazard ratios (HRs) (Cox proportional hazards regression model), with adjustment for clinical and pathological factors. All tests were two-sided, and a *P* value of less than 0.05 was considered statistically significant. In order to take into account the multiple testing that was performed, the final *P*-values are adjusted to control for a False Discovery Rate (FDR) of 5% [19]. All variables significant at *P* = 0.05 (after adjustment for polymorphisms) were included in the multivariate models. Statistical analysis was carried out using SAS V9.2 (Cary, NY).

Three populations were defined for analysis: for the primary response criteria, this included all eligible patients with tumoral evaluation by scan and review at six months, for toxicity this included all patients receiving at least one dose of the FOLFIRI® + B treatment and for survival, this included all eligible patients without major protocol deviations.

## Results

### Patient characteristics

Sixty-two patients were enrolled in this trial between January 2007 and August 2009 (Table 1). One patient who had been treated 2 years earlier for a squamous cell vocal cords tumor was considered a major protocol violation and excluded from the survival and response analyses. Two other patients were not evaluable for the primary response endpoint (one was lost to follow-up before the CT scan evaluation; the second stopped treatment due to a cause not related to the trial). They all received at least one dose of the FOLFIRI® + B treatment and are included for toxicity analyses.

**Table 1:** Patient and tumor characteristics at baseline (N = 62)

	***N***	**(%)**
Age (years)		
Median	67.9	
Range	60.4–75.4	
Sex		
Male	25	(40.3)
Female	37	(59.7)
ECOG performance status		
0	20	(32.3)
1	39	(62.9)
2	3	(4.8)
Primary tumor location		
Colon	53	(84.5)
Rectum	9	(14.5)
Metastases		
Liver	54	(87.1)
Lung	28	(45.2)
Lymph nodes	16	(25.8)
Peritoneum	17	(27.4)
Others	14	(22.5)
Number of organs involved (measurable)		
1	8	(12.9)
2	12	(19.3)
≥ 3	42	(66.1)
Adjuvant chemotherapy	17	(27.4)
Radiotherapy	8	(12.9)
Surgery	50	(80.6)

### Response and survival

**Figure 1:**
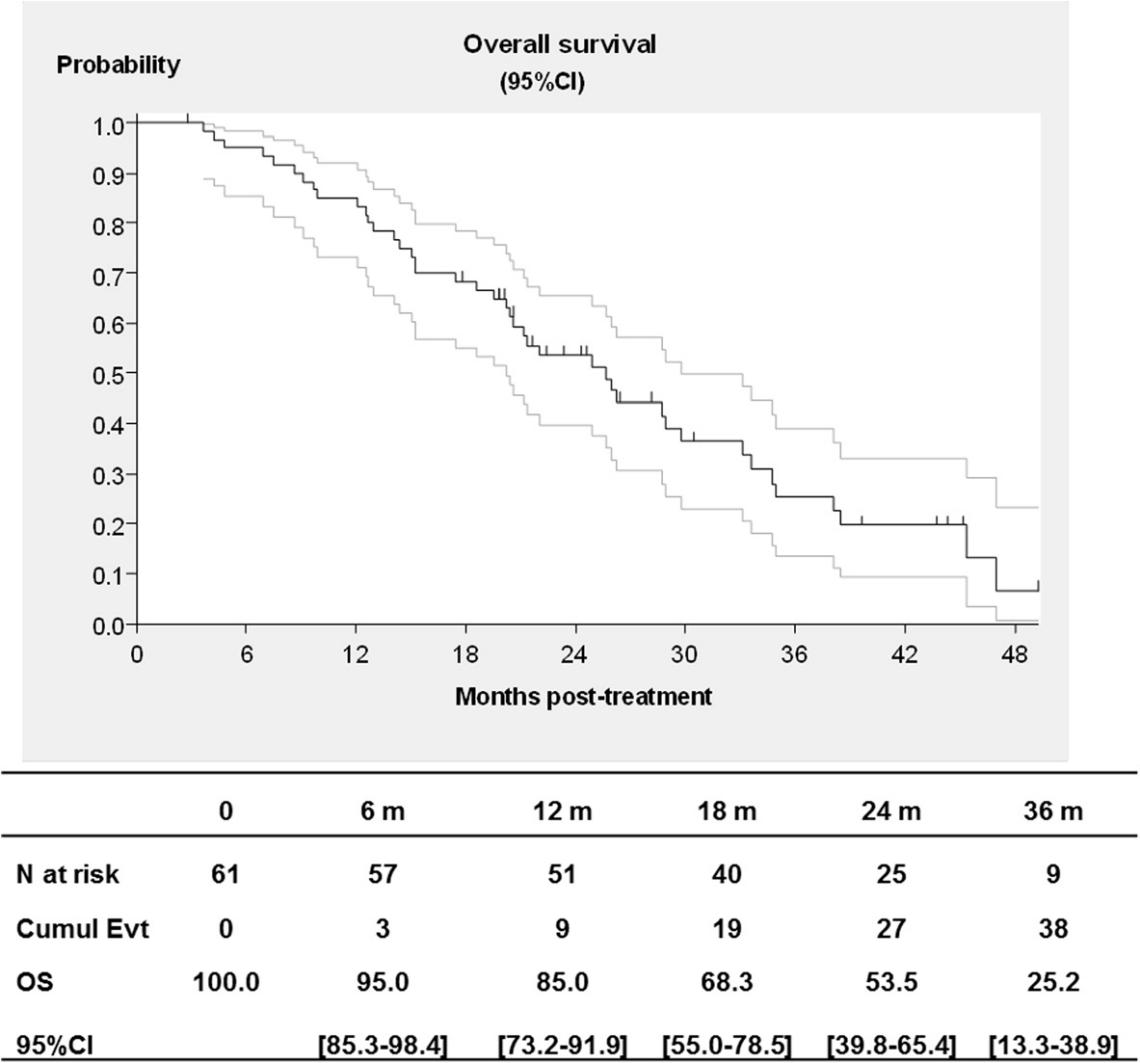
Overall survival for colorectal cancer patients treated with FOLFIRI® and bevacizumab in first-line treatment (with 95% confidence interval, CI).

**Figure 2:**
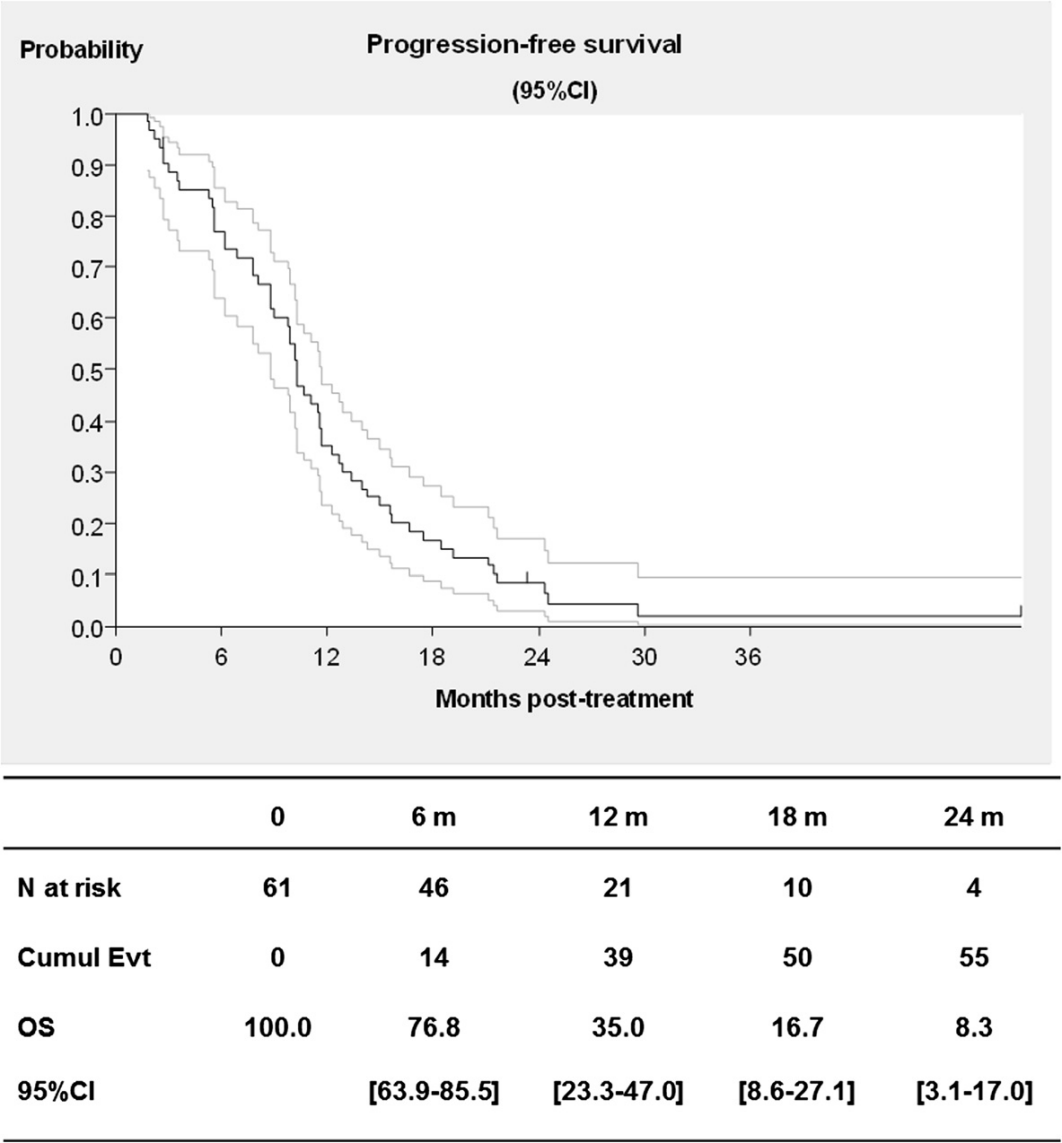
Progress-free survival for colorectal cancer patients treated with FOLFIRI® and bevacizumab in first-line treatment (with 95% confidence interval, CI).

At the cut-off date of December 2011, median follow-up was 43.6 months (range: 26.3–45 months) and no patient was still receiving treatment. Responses were evaluated at 6 months for 59 eligible and assessable patients, with 28 patients achieving a partial response (objective response rate 47.5%, 95% CI; 34.3-60.9), 20 with stable disease (33.9%), and 11 with progressive disease (18.6%). The median duration of response was 9.5 months (range: 2.7-20). Median OS was 25.7 months (95% CI; 20.2-29.7) and median PFS was 10.3 months (95% CI; 8.8-11.7). At one year, survival was 85% (95% CI; 73.2-91.9%), and PFS was 35% (95% CI; 23.3-47]) (Figures 1–2). Eleven patients could subsequently be resected following treatment.

### Tolerance

Chemotherapy administration was generally compliant. Relative dose intensity was higher than 90% for each drug (Irinotecan 94.5%, FU 93.8% and bevacizumab 94.5%). A total of 1058 cycles were administered (median 13, range 3–62) with median cumulative doses of 2200.5 mg/m^2^ for irinotecan, 34.624 g/m^2^ for FU and 60.9 mg/kg for bevacizumab. Cycle delays (i.e. delay in schedule ≥ 8 days) were observed for 28 patients (43.5%), for toxicity in all cases but one (mainly hematological or diarrhea). Five patients (8%) stopped treatment for toxicities. Fourteen patients (22.6%) stopped therapy for surgical procedures. Nine serious adverse events linked to treatment were observed for seven patients (11.3%): (3 diarrhea, 2 colitis, 1 renal failure, 1 gastritis, 1 phlebitis, and 1 leukocytes).

All 62 patients were assessable for toxicity. No toxic deaths occurred (Table 2). A toxic effect (of any grade) led to dose modifications for 32 patients (51.6%) over the first 20 cycles of treatment.

**Table 2:** Drug-related toxicity per patient for colorectal cancer patients treated with FOLFIRI® and bevacizumab in first-line treatment (n = 62)

	NCI-CTCAE* Grade 1–2		**NCI-CTCAE Grade 3–4**	
	***N***	**(%)**	***N***	**(%)**
Neutropenia	11	(17.7)	10	(16.1)
Febrile neutropenia	0	–	0	–
Anemia	4	(6.5)	0	–
Thrombocytopenia	1	(1.6)	0	–
Nausea	20	(32.3)	1	(1.6)
Vomiting	6	(9.7)	0	–
Diarrhea	19	(30.6)	7	(11.3)
Stomatitis/mucositis	16	(25.8)	2	(3.2)
Neurosensory	1	(1.6)	1	(1.6)
Asthenia	3	(4.8)	4	(6.4)
Gastrointestinal perforation	0	–	0	–
Hypertension	1	(1.6)	0	–
Venous thromboembolism	3	(4.8)	0	–
Proteinuria	8	(12.9)	0	–
Bleeding	7	(11.3)	0	–
Alopecia	10	(16.1)	0	–

*NCI-CTCAE - National Cancer Institute Common Terminology Criteria for Adverse Events v3.0.

### Genotyping

**Figure 3:**
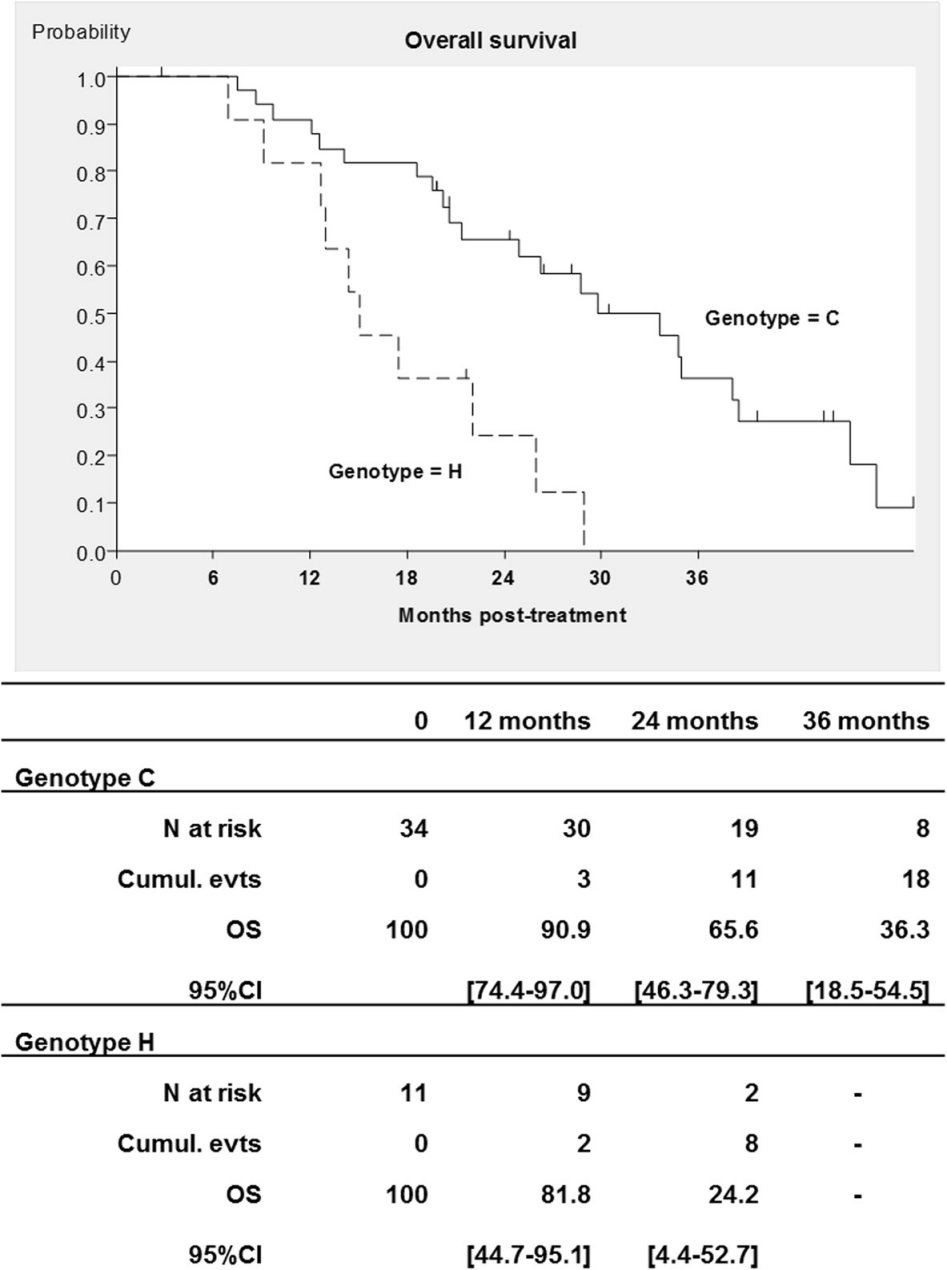
Overall survival for genotypes C and H of the rs25648 polymorphism for colorectal cancer patients with FOLFIRI® and bevacizumab in first-line treatment (with 95% confidence interval, CI).

Fifty eight patients (94%) agreed to participate in the pharmacogenetic study and material was received for 46 patients. One major protocol exclusion was observed leaving 45 eligible patients for genotyping. The detailed results of genotyping are presented in Additional file 1. No significant deviations from the Hardy-Weinberg equilibrium were noticed for any. Concerning the *VEGFA* genotype, it was possible to detect a significant linkage disequilibrium between the two promoter polymorphisms, but the exonic SNP (rs25648) was not in linkage disequilibrium with the two other SNPs. Concerning the *TYMS* genotype, there was a linkage disequilibrium between the 3′UTR insdel variation and the 5′UTR tandem repeat, which was of borderline significance. After correction for multiple testing, not one of the polymorphisms studied was associated with toxicity or PFS but the S178S synonymous variation (rs25648) was significantly associated with OS (rough *P*-value = 0.0013, corrected *P*-value = 0.0104, HR = 0.605, 95% CI, 1.57-8.30) (Figure 3). Looking back at its association with PFS, it appeared significant (*P*-value = 0.049) only before correction for multiple testing.

In univariate analysis *VEGFA* rs25648 polymorphism, sex, serum alkaline phosphatase (ALP) and lactate dehydrogenase (LDH) were significantly associated with OS. In multivariate analysis, sex (*P* = 0.0136), serum ALP (*P* = 0.0085) and the *VEGFA* rs25648 polymorphism (*P* = 0.0041) remained significant (Table 3).

**Table 3:** Univariate and multivariate analyses for demographic, clinical and genetic data with overall survival for colorectal cancer patients treated with FOLFIRI® and bevacizumab (n = 45)

	**Univariate**			**Multivariate**	
	***N *** **(45)**	**HR [95% CI]**	***P***	**HR [95% CI]**	***P *** **(Wald)**
Sex: Male	16 (35.6)	ref.		ref.	ref.
Female	29 (64.4)	0.43 [0.20; 0.90]	0.02	0.38 [0.18; 0.82]	0.01
Age, y: <=65	20 (44.4)	ref.			
> 65	25 (55.6)	1.09 [0.52; 2.28]	0.81	-	-
ECOG/PS: 0	16 (35.6)	ref.			
1-2	29 (64.4)	1.26 [0.60; 2.70]	0.55	-	-
Primary tumor: Colon	28 (62.2)	ref.			
Rectum	17 (37.8)	1.16 [0.56; 2.40]	0.69	-	-
Non-mucinous: No	5 (11.1)	ref.			
Yes	40 (88.9)	0.45 [0.15; 1.36]	0.15	-	-
Metastatic sites: 1	13 (28.9)	ref.			
>1	32 (71.1)	0.77 [0.36; 1.65]	0.50	-	-
Liver-only metas.: No	33 (73.3)	ref.			
Yes	12 (26.7)	1.14 [0.52; 2.52]	0.74	-	-
Resected primary tumor: No	10 (22.2)	ref.			
Yes	35 (77.8)	0.67 [0.29; 1.51]	0.33	-	-
Previous adjuvant CT*: No	34 (75.6)	ref.			
Yes	11 (24.4)	0.85 [0.36; 1.98]	0.70	-	-
High ALP^†^: No	23 (51.1)	ref.		ref.	ref.
Yes	19 (42.2)	2.80 [1.25; 6.26]	<0.009	4.21 [1.44; 12.31]	0.008
High LDH^§^: No	16 (35.6)	ref.			
Yes	13 (28.9)	4.73 [1.42; 15.81]	<0.006	1.46 [0.62; 3.43]	0.39
High ACE^††^: No	17 (37.8)	ref.			
Yes	22 (48.9)	2.07 [0.91; 4.72]	0.08	-	-
rs25648: C	34 (75.6)	ref.		ref.	ref.
H/V	11 (24.4)	3.605 [1.57;8.30]	0.01**	3.58 [1.50; 8.57]	0.004
rs2010963: C	17 (37.8)	ref.			
H/V	28 (62.2)	1.228 [0.59;2.56]	0.58	-	-
rs699947: C	34 (75.6)	ref.			
H/V	11 (24.4)	1.324 [0.59;2.99]	0.50	-	-
rs8175347: C	15 (33.3)	ref.			
H/V	30 (66.7)	0.632 [0.30;1.35]	0.23	-	-
3′UTR: C	23 (51.1)	ref.			
H/V	22 (48.9)	1.298 [0.64;2.63]	0.47	-	-
5′UTR: V	11 (24.4)	ref.			
C/H	33 (73.3)	0.972 [0.44;2.13]	0.94	-	-
5′UTR: C	13 (28.9)	ref.			
H/V	31 (68.9)	0.886 [0.41;1.91]	0.76	-	-

*CT = chemotherapy.

**P-value corrected to false discovery rate of 5% [19].

^†^ALP = alkaline phosphatase, Missing: 3 (6.7).

^§^LDH = lactate Dehydrogenase, Missing: 16 (35.6).

^††^ACE = angiotensin-converting Enzyme, Missing: 6 (13.3).

## Discussion

The primary objective of this trial was to assess the objective response rate after treatment with FOLFIRI® + B in metastatic CRC. We observed 47.5% objective partial responses with no complete responses at six months. These results appear slightly higher than the most recent phase III trial report with 142 patients showing a response rate of 40.1% [20], similar to as reported by Souglakos et al. [21] (45.5%), and higher than reported in a recent phase III trial (36.8% [22], although they are lower than in previous studies (eg. BICC-C [5] or Kopetz et al. [23]) with 59% and 65% objective responses respectively.

With a median follow-up time of 43.6 months, the median PFS was 10.3 months, and one-year PFS was 35%. This supports previous reports after FOLFIRI® + B treatment with PFS reported between 9 [24] to 11.6 months [25], and 12.8 months [23]. The median OS in the present trial was 25.7 months, with a one-year survival of 85%. Once again, these rates support previously and recently published rates with OS reported between 22 months [22] 23.7 months [25], 25.7 [21], 28 months (one-year OS of 87%) [26] and 31.3 months [23]. In a recent observational report involving over 240 patients, median PFS was reported at 10.2 months and median OS at 25.5 months, once again providing further support for these patterns [27]. Our results show that a curative hepatic surgery could be carried out for 11 patients (18.6%) that were judged to be unresectable before FOLFIRI + B treatment. The rate of hepatic surgery with FOLFIRI® + B was not reported in the BICC-C study [5,26], and it was 6.5% in the Beat Study Cohort and 9.3% in Kopetz et al.’s study [23].

In randomized trials in patients with metastatic CRC, bevacizumab has been shown to improve response rates, OS and PFS when combined with chemotherapy regimens like bolus FU/LV [28], irinotecan plus bolus FU/LV (IFL) [6] and oxaliplatin plus infusional FU/LV (FOLFOX) [29]. In a systematic review for patients with advanced CRC receiving first- or second-line fluoropyrimidine-based chemotherapy, the addition of bevacizumab improved PFS and OS, although toxicity was also increased [30]. A more recent systematic review and meta-analysis including over 3000 patients from randomized trial [31] shows a distinct advantage for PFS when bevacizumab is added. In subgroup analyses, the effect was strongest for FU- and irinotecan-based chemotherapy regimens and less marked in oxaliplatin-based regimens.

Severe toxic effects were mainly hematologic and less frequently gastrointestinal. The rate of grade ≥3 toxicities was low. At 19.4%, neutropenia was the most frequent severe toxicity, as has been described in other trials [20], although the rate was lower than described in other studies at (53.6%) [5] or (40%) [23]. The rate of severe diarrhea (11.3%) was similar to the BICC-C study (10.7%) [5] and higher than in Kopetz’s study (2%) [23]. Considering adverse events which could be related to bevacizumab, we had no gastrointestinal perforation compared to 0% [5,23] to 2% [25] reported in the literature; no severe bleeding compared to 0% [5,23] -3% [25], no Grade 3/4 proteinuria, similar to 1% in Van Cutsem et al. [25]; no severe hypertension, lower than reported in the literature at 5% [25] to 12.5% [5] and 19% [23]; and 1.6% Grade 3/4 venous thromboembolism events compared to 1% [25] and 19% [23]. It should be noted that for the 11 patients who were operated on in this trial, no delayed wound healing was noted, with bevacizumab being stopped six weeks before surgery.

Associations between *TYMS* polymorphisms and FU efficacy and toxicity in CRC have been reported in several studies [32-35] but not all [36]. In our study, no influence of *TYMS* polymorphisms on the efficacy or toxicity of the treatment was demonstrated. The limited size of the population tested as well as the fact that the patients did not receive exclusively FU probably explain the lack of associations. The toxicity of irinotecan has been associated with a polymorphism of the SN-38-detoxifying enzyme, *UGT1A1*, located in the promoter of the gene [37]. However, the risk of experiencing irinotecan-induced hematological toxicity appears to be a function of the dose administered [37]; the risk is higher at the dose usually prescribed in the US (340 mg/m^2^ every 3 weeks) than at the dose prescribed in Europe (180 mg/m^2^ every two weeks). This may also explain the lack of associations in our study.

We found an association between OS and the presence of a polymorphic exonic synonymous variation in the *VEGFA* gene. Interestingly, the rs25648 variation is one of the three SNPs influencing VEGF serum levels [38], as well as *VEGFA* mRNA levels in colorectal adenocarcinoma [39]. One could hypothesize that the higher levels of *VEGFA* produced by the variant allele limits the efficacy of the anti-VEGF antibody and, therefore, has an impact on patient survival. However, the association of this polymorphism with outcome was only significant for OS and not for PFS, which would indicate a general prognostic impact rather than a predictive role for bevacizumab efficiency. Studies involving a larger number of patients should be undertaken with a special focus on this polymorphism which has not been studied extensively up to now.

The fact that a synonymous polymorphism in the coding sequence of *VEGFA* exerts an effect on VEGF levels in plasma may be explained by a difference in the 3-dimension structure or the half-life of the transcribed mRNA, which may introduce differences in its handling by the translation machinery. Alternatively, the difference in tRNA availability because of the use of a rare codon may lead to a decrease in translation rate, and thus to a reduced production of the protein.

Some limits should be taken into account when interpreting this data. Firstly, the non-comparative nature of this non-randomized phase II trial should be kept in mind when comparing efficacy results. It should also be noted that although we found a significant effect for the primary endpoint of objective response, no complete responses were observed.

## Conclusions

The present results support the growing body of evidence from phase II [24], phase III and observational studies indicating that FOLFIRI® + B is an active and safe treatment for first-line treatment of metastatic colorectal cancer, with almost half of patients showing an objective response and comparatively long median OS. Further, almost 1/5 initially unresectable patients became resectable after treatment, offering potential for longer survival. It has a good safety profile, with relatively low rates of thromboembolism compared to other alternative chemotherapy associations. The association between the genetic polymorphism rs25648 and improved OS is encouraging, but needs to be confirmed in further trials.

## Abbreviations

CRC: Colorectal cancer; VEGF: Vascular endothelial growth factor; FOLFIRI + B: Irinotecan, leucovorin, fluororacil + bevacizumab; FU: Infusional 5-fluorouracil; OS: Overall survival; PFS: Progression-free survival; RECIST: *Response Evaluation Criteria In Solid Tumors*; DNA: Deoxyribonucleic acid.

## Competing interests

The authors declare that they have no competing interests.

## Authors’ contributions

YB conceived of the study and drafted the manuscript. CL and SMP participated in the design and coordination of the study and statistical analysis. MP participated in the design of the study, performed the statistical analysis and helped to draft the final manuscript. RB, PT and LC recruited patients to the study. VLM and SA carried out the molecular genetic studies and participated in the sequence alignment. DB, RBr and MF participated in the design of the study. JR carried out the molecular genetic studies, participated in the sequence alignment, and drafted the manuscript. All authors read and approved the final manuscript.

## Supplementary Material

Additional file 1 Distribution of the genotypes of the 6 polymorphisms determined in 46 patients for colorectal cancer patients treated with FOLFIRI® and bevacizumab in first-line treatment. Click here for file
