# Supplementary material for: FOLFIRI® and Bevacizumab in first-line treatment for colorectal cancer patients: safety, efficacy and genetic polymorphisms
Source: BMC Res Notes. 2014 Apr 23;7:260. doi: 10.1186/1756-0500-7-260 (PMC4022139; doi:10.1186/1756-0500-7-260)
Supplement: Additional file 1 — Distribution of the genotypes of the 6 polymorphisms determined in 46 patients for colorectal cancer patients treated with FOLFIRI® and bevacizumab in first-line treatment. [file 1756-0500-7-260-S1.doc]

**Appendix A:**

Distribution of the genotypes of the 6 polymorphisms determined in 46 patients for colorectal cancer patients treated with FOLFIRI® and bevacizumab in first-line treatment

| Patient | ***VEGFA*** | | | ***UGT1A1*** | ***TYMS*** | |
| --- | --- | --- | --- | --- | --- | --- |
|  | rs2010963 | rs25648 | rs699947 | rs8175347 | 3'UTR | 5'UTR |
| 1 | G/G | C/C | A/C | 6/7 | +6/-6 | 2C/3G |
| 2 | G/G | C/C | A/A | 6/7 | +6/-6 | 2C/3G |
| 3 | C/G | C/C | A/C | 6/7 | +6/-6 | 2C/3G |
| 4 | C/G | T/C | A/C | 6/7 | +6/-6 | 2C/3C |
| 5 | C/G | T/C | A/C | 6/6 | +6/+6 |  |
| 6 | C/G | T/C | A/C | 7/7 | +6/+6 | 2C/3G |
| 7 | G/G | C/C | A/A | 7/7 | +6/+6 | 3C/3G |
| 8 | C/G | C/C | A/C | 6/7 | -6/-6 | 3C/3C |
| 9 | G/G | C/C | A/A | 6/6 | -6/-6 | 3C/3G |
| 10 | C/G | C/C | A/C | 7/7 | -6/-6 | 3G/3G |
| 11 | G/G | C/C | A/C | 6/7 | +6/+6 | 2C/2C |
| 12 | C/G | C/C | A/C | 6/6 | +6/+6 | 2C/3C |
| 13 | C/G | C/C | A/C | 7/7 | +6/+6 | 2C/3C |
| 14 | C/C | C/C | C/C | 6/6 | +6/+6 | 2C/3C |
| 15 | C/G | C/C | C/C | 6/6 | +6/+6 | 2C/2C |
| 16 | G/G | C/C | A/A | 6/6 | +6/-6 | 2C/3G |
| 17 | C/G | C/C | C/C | 6/7 | +6/-6 | 3C/3G |
| 18 | G/G | C/C | A/C | 7/7 | +6/+6 | 3G/3G |
| 19 | G/G | C/C | A/C | 6/7 | +6/+6 | 2C/3G |
| 20 | G/G | T/C | A/A | 6/6 | +6/+6 | 2C/2C |
| 21 | C/G | T/C | A/C | 6/6 | +6/+6 | 3C/3C |
| 22 | C/G | C/C | A/C | 7/7 | +6/+6 | 2C/2C |
| 23 | C/C | C/C | C/C | 6/6 | +6/-6 | 2C/2C |
| 24 | G/G | T/C | A/A | 7/7 | +6/-6 | 2C/3G |
| 25 | C/C | C/C | C/C | 6/6 | +6/+6 | 2C/2C |
| 26 | C/G | T/C | A/C | 6/6 | +6/-6 | 2C/2C |
| 27 | G/G | C/C | A/C | 6/7 | +6/+6 | 2C/3C |
| 28 | C/G | C/C | A/C | 6/6 | +6/+6 | 2C/2C |
| 29 | C/G | C/C | A/C | 6/6 | +6/+6 | 3C/3C |
| 30 | G/G | C/C | A/C | 6/7 | +6/+6 | 3C/3C |
| 31 | C/G | C/C | A/C | 6/6 | +6/-6 | 2C/3C |
| 32 | C/G | C/C | C/C | 6/7 | +6/-6 | 2C/3G |
| 33 | G/G | C/C | A/C | 6/7 | -6/-6 | 2C/3G |
| 34 | C/G | C/C | C/C | 6/6 | +6/-6 | 2C/3G |
| 35 | C/G | C/C | C/C | 6/7 | +6/-6 | 2C/3G |
| 36 | C/G | C/C | A/C | 6/7 | +6/-6 | 2C/3G |
| 37 | G/G | C/C | A/A | 6/6 | +6/+6 | 2C/2C |
| 37 | C/G | C/C | C/C | 6/7 | +6/+6 | 3C/3C |
| 38 | C/G | C/C | A/C | 6/6 | +6/-6 | 3C/3G |
| 39 | G/G | T/C | A/C | 6/7 | +6/+6 | 2C/3C |
| 40 | C/G | T/C | A/C | 6/7 | +6/-6 | 2C/3G |
| 41 | C/G | C/C | C/C | 7/7 | +6/+6 | 2C/2C |
| 42 | C/C | C/C | C/C | 6/7 | +6/+6 | 2C/2C |
| 43 | G/G | T/C | A/C | 6/7 | +6/-6 | 3C/3G |
| 44 | C/C | C/C | C/C | 6/7 | +6/-6 | 2C/3G |
| 45 | G/G | T/C | A/A | 6/7 | +6/-6 | 3C/3G |
